# Methylmercury Contamination of Laboratory Animal Diets

**DOI:** 10.1289/ehp.7816

**Published:** 2005-04-20

**Authors:** Bernard Weiss, Sander Stern, Elsa Cernichiari, Robert Gelein

**Affiliations:** Department of Environmental Medicine, University of Rochester School of Medicine and Dentistry, Rochester, New York, USA

**Keywords:** animal feed, laboratory diets, methylmercury, rats

## Abstract

In the midst of research focusing on the neurodevelopmental effects of mercury vapor in rats, we detected significant levels of mercury (30–60 ng/g) in the blood of nonexposed control subjects. We determined that the dominant form of the mercury was organic and that the standard laboratory chow we used in our vivarium was the source of the contamination. The dietary levels were deemed of potential biologic significance, even though they might have fallen below the limits of measurement specified by the supplier. All investigators employing animals in research must assess such potential contamination because dietary agents may alter *a*) conclusions based on intentionally administered doses, *b*) outcomes by interacting with other agents that are the primary focus of the research, and *c*) outcomes of research unrelated to the toxic effects of experimentally administered agents.

Methylmercury is recognized as a potent poison, especially for its neurotoxic properties (Davidson et al. 2004). We report here that diets commonly employed in laboratory animal research may contain concentrations of organic mercury, methylmercury most likely, that are sufficient to directly affect the results. Our concerns are 2-fold. First, research focusing on methylmercury effects will include control data contaminated by nonzero exposure levels, and exposure concentrations for detected effects in “exposure groups” will differ from dose levels measured in the intentionally administered agent. Use of such data could compromise conditions for setting adequate exposure standards. Second, investigations not focusing on methylmercury directly, for example, studies of polychlorinated biphenyls (PCBs), which interact with methylmercury (Grandjean et al. 2003; Stewart et al. 2003), might inadvertently include control baselines determined partially by exposures to methylmercury. In such instances, treatment-group comparisons may be distorted by such effects. And, experiments directly aimed at combined PCB–methylmercury effects (e.g., Widholm et al. 2004) might produce confusing outcomes.

## Methods and Results

The data described in this article are the byproducts of an investigation we undertook to study the developmental neurotoxicity of mercury vapor in rats. We did not *a priori* plan the diet assay protocols reported here, and although limited, the results of these evaluations have significance that must be considered in both evaluating past studies and designing future ones. Because surprisingly little is known about the developmental effects of metallic mercury despite its lengthy history in toxicology and its recognized potency as a neurotoxicant (Clarkson 2002), we had planned to examine this aspect of it.

In experiment 1, female Long-Evans rats (Charles River, Wilmington, MA) were bred 3 weeks after receipt from the supplier and then exposed via inhalation to mercury vapor concentrations of 0, 30, 100, or 300 μg/m^3^ during gestational days (GD)6–20. The 0-ppm control group was held in a separate mercury-free chamber during exposures. The mercury vapor concentration within a chamber was monitored continuously by a continuous mercury vapor analyzer dual-beam ultraviolet photometer in standard flow configuration (model 791.741; EPM Environmental Products Manufacturing, Dalerstraat, the Netherlands), which was capable of measuring concentrations from 2 to 1,999 μg/m^3^ in air. Mercury in the blood served as a biomarker of exposure. A cold vapor atomic absorption procedure (Lapham et al. 1995; Magos and Clarkson 1972) was used to assay blood samples from the pregnant dams on GD18 and from the pups on postnatal day (PND)4 and PND18.

**Figure 1:**
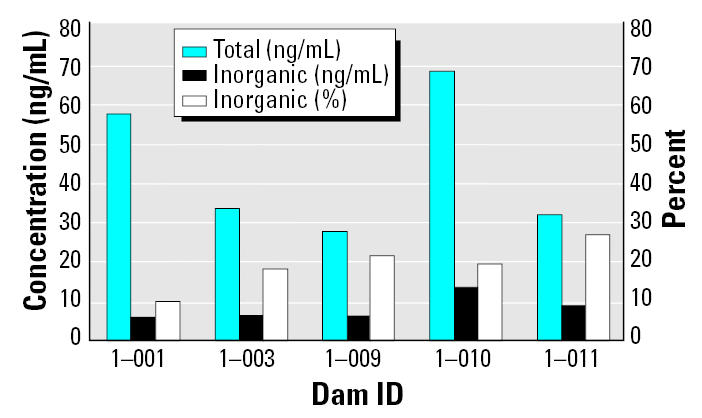
Blood levels of total mercury, inorganic mercury, and percent inorganic mercury in control dams. The inorganic component is the product of the slow conversion of methylmercury, the source of the mercury, to the inorganic form (e.g., Rowland et al. 1984).

Control dam (Figure 1) and pup (Table 1) samples showed unexpected, relatively high levels of mercury (particularly as organic mercury). By analyzing the samples for the presence of inorganic mercury specifically, we could estimate the amount of organic mercury (i.e., total – inorganic). As shown in Figure 1 and Table 1, the blood values were predominantly of the organic form.

When we first detected the high levels of mercury in our control subjects, we immediately sought to evaluate, on a probing basis, potential sources. Our sampling procedures were designed and employed to prevent and mitigate recognized potential sources of contamination, as we have done in the past. We did not detect mercury in either the control chamber or the room housing the chambers; in either the atmosphere in the vivarium room assigned to the animals in the experiment or the bedding in the animal cages; in the breath of the investigators who pipetted the blood during the tail-nick procedure used with the dams; or in the heparin that was used for the collection procedures. We did not believe our mercury assay procedures were at fault because they are continually evaluated as part of an international mercury quality control program administered by the Centre de Toxicologie du Québec (Institute National de Santéé Publique, Sainte-Foy, Québec, Canada), which has run the Interlaboratory Comparison Program since 1979.

Together, these results led us to suspect the diet as the source of contamination. Purina Laboratory Rodent Diet 5001 (Scott’s Distribution, Hudson, NH), which has widespread use, was fed to the rats in this research. Sample pellets from the batch in use at that time were ground or milled and then analyzed (we used more than one procedure to systematically replicate our observations and convince ourselves that we had not introduced confounds). For the second procedure, we used a ball mill with zirconium pellets. Between samples, both were washed, and then the jar and Zr pellets were baked at 150°C for several hours to ensure the absence of mercury. Then the samples were individually ground for at least 48 hr. These analyses, as shown by the examples in Table 2, verified that the elevated mercury levels in our control dams and pups were due to the contaminated diet and that they reflected organic mercury.

The Purina 5001 diet is an open diet; that is, its ingredients are subject to change, depending on the source of the raw materials. Fish meal is one of the ingredients, and it is possible that methylmercury present in tuna scraps, for example, may have been the source of the fish meal used in the batch provided by the vivarium. The supplier gives the limit of detection as 0.02 ppm (20 ng/g), so the problem apparently escaped detection. Even so, such levels are excessively high for experiments on mercury, especially those focusing on low-level dose–response outcomes. We were unprepared for the results in the present study because, in an earlier methylmercury study with mice (Stern et al. 2001) also fed the Purina 5001 diet, we detected no mercury in control dams or pups.

To preclude contamination in further experiments, we contacted BioServ (Frenchtown, NJ), a supplier of laboratory animal feed, which recommended the synthetic AIN-93G diet. The protein in this diet is casein. BioServ provided samples of whole pellets as well as the casein incorporated into the diet. Table 3 shows the results of our analysis of the ground pellets and, independently, of the casein. Although the pellets contained mercury, it was 100% inorganic. To determine its effects on blood levels, we fed three females the AIN-93G diet and three males the Purina 5001 diet. We detected no mercury in blood samples from the females fed the AIN-93G diet, but we did find it in the males fed the Purina 5001 diet (28.24, 22.84, and 16.08 ng/mL). (Only total mercury was measured because the focus was on comparing mercury levels in the AIN-93G diet-fed subjects with those fed Purina 5001.) Because inorganic mercury is poorly absorbed after ingestion, these findings are not surprising. These results also confirmed that the rats did not carry a significant mercury burden when they were received from the supplier.

More recently, in our ongoing attempts to find a suitable, mercury-free diet, we analyzed samples of the Teklad 2018 diet (Harlan Teklad, Madison, WI), which does not contain fish meal. We ground four pellets in a mortar to obtain a fine powder, which was then digested with sulfuric acid. No mercury was detected.

## Discussion

Figure 1 shows why the possibility of methylmercury contamination in laboratory animal diets cannot be ignored. The levels in control dams were close to the 58 ng/g determined by the National Academy of Sciences committee on methylmercury, on the basis of developmental neurotoxicity, as the benchmark dose lower bound for cord blood in human populations (National Research Council 2000). Although not measured here, we would certainly expect fetal levels in our rats to be even higher (Watanabe et al. 1999), especially in brain, because levels in rodent neonates fall rapidly after birth (Newland and Reile 1999; Stern et al. 2001).

It is impossible to know how much of the published experimental data, as well as ongoing research, may be distorted by contaminated diets. Although the “certified” diets provided by manufacturers may prove useful to investigators, independent confirmation of ingredients should be encouraged. Biomarkers of exposure, that is, tissue indices, are the key to interpreting exposure data. Such direct measures in experimental subjects (including controls) provide assurances that the investigator’s protocols are properly conducted. We uncovered our problem only because we include blood and tissue assays in our standard operating procedures when conducting research with mercury. We strongly urge all researchers to do likewise. In a brief survey of recent literature, we have been surprised by how often researchers neglect to mention diet, or describe it in terms such as “standard rat chow.” Infrequently, the authors may provide the name of the supplier and the diet label, which should be the minimum information provided.

Although the results reported here stem from our research focusing on mercury, the issue of diet-based contamination certainly is not limited to one agent. For example, investigators who study endocrine disruptors have become concerned by the presence of agents in laboratory animal diets that may mimic estrogens (Boettger-Tong et al. 1998). Particularly in investigations of low-level, environmentally relevant exposures, diet is an unwelcome confounder.

## Figures and Tables

**Table 1:** Blood mercury in control pups from dams that had been fed the Purina 5001 diet (experiment 1).

		Mercury	
Litter ID	Age	Total (ng/mL)	Inorganic (%)
1-001-1D1	PND4	15.5	ND
1-003-1D1	PND4	18.3	ND
1-009-1D1	PND4	11.1	ND
1-010-1D1	PND4	11.5	ND
1-011-1D1	PND4	14.6	ND
1-001-11	PND18	5.3	62
1-003-11	PND18	3.8	87
1-009-11	PND18	3.2	ND
1-010-11	PND18	3.3	ND
1-011-11	PND18	4.1	ND

ND, not detected (the detection limit in our laboratory is 0.75 ng Hg). Samples were pooled within litters to provide a volume adequate for the assays. By PND18, mercury levels had declined substantially (compare Newland and Reile 1999; Stern et al. 2001).

**Table 2:** Total mercury in rat chow samples.

	Mercury	
Pellet/method	Total (ng/g)	Inorganic (%)
Purina 5001		
Ground	57.9	0
	30.1	48
	27.6	31
	15.3	
	12.0	
	6.7	
Homogenized	33.0	
	8.6	
	18.0	
	12.0	
Harlan Teklad 2018	ND	
	ND	

ND, not detected. The percentage of inorganic mercury, determined only for the first three ground pellet samples, indicated significant organic mercury contamination.

**Table 3:** Mercury content analysis of BioServ AIN-93G diet and casein.

	Mercury	
Sample	Total (ng/g)	Inorganic (%)^a^
AIN-93G		
1	317.9	
2	191.5	
3	223.8	
4	182.6	
5	85.1	
6	96.9	100
7	62.9	100
8	71.8	100
9	123.3	100
10	117.7	100
11	144.8	100
Milled sample^b^	77.98	
Milled sample^b^	110.20	
Ground sample^b^	122.78	
Ground sample^b^	38.04	
Milled sample^c^	139.35	
Ground sample^c^	54.76	
Mean	127.14	
Casein		
1	ND	
2	ND	
3	ND	
4	ND	

ND, not detected. Although variability in total mercury across samples was large, organic mercury was consistently absent.

a Determined for only samples 6–11.

b Samples were digested normally with sodium hydroxide and cysteine and then collected on silver traps to detect the presence of mercury.

c Samples were dissolved in 10% nitric acid and then collected on silver traps to detect the presence of mercury.
